# Exploring the Impact of Ultrasonic Activation on the Antimicrobial Activity of Root Canal Sealers: A Year‐Long Ex Vivo Investigation Into Bacterial Survival

**DOI:** 10.1111/iej.70227

**Published:** 2026-07-22

**Authors:** F. L. P. Viana, M. A. F. Damasceno, M. P. Alcalde, R. R. Vivan, I. G. de Moraes, M. A. H. Duarte, E. T. Pinheiro, B. C. Vasconcelos

**Affiliations:** ^1^ Post‐Graduate Program in Dentistry Federal University of Ceará Fortaleza Brazil; ^2^ Department of Dentistry, Endodontics and Dental Materials, Bauru Dental School University of São Paulo (USP) Bauru SP Brazil; ^3^ Department of Dentistry, São Paulo School of Dentistry University of São Paulo (USP) São Paulo Brazil

**Keywords:** bacterial biofilm, endodontic sealers, endodontics, *Enterococcus faecalis*, *Streptococcus oralis*, ultrasound

## Abstract

**Aim:**

This study investigated the long‐term survival of bacteria in root canals obturated using two endodontic sealers—AH Plus Jet (AHP) and BioRoot RCS (BCS)—while also assessing the influence of ultrasonic agitation on their antimicrobial effectiveness.

**Methodology:**

One hundred human single‐rooted teeth were contaminated for 21 days with dual‐species biofilms of *Enterococcus faecalis* and *Streptococcus oralis* . Samples were collected at various treatment stages: initial (S1), post‐instrumentation (S2), and after filling removal (S3), with additional samples after 7 days (S4). Groups were categorized by sealer type, incubation duration (30 days and 12 months), and ultrasonic agitation (UA): AHP, AHP/UA, BCS, BCS/UA, and a control group (CON) without a sealer. Bacterial survival was assessed through colony‐forming unit (CFU) counts, and viability was visualized via confocal laser scanning microscopy (CLSM).

**Results:**

Bacterial counts immediately after root filling removal (S3) remained comparable to those observed before obturation (S2) but significantly increased after 7 days of nutritional exposure (S4) in all groups (*p* < 0.05). CLSM corroborated the CFU findings, indicating that bacteria remained viable within dentinal tubules after 30 days and 12 months. The use of UA enhanced the antimicrobial effectiveness of the sealers during the 30‐day period (*p* < 0.05). After 12 months, AHP exhibited lower bacterial growth, particularly for *E. faecalis* , compared with BCS (*p* < 0.05).

**Conclusions:**

Ultrasonic agitation reduced the ability of residual bacteria to recolonize the root canal space during the initial 30‐day period. However, viable bacteria persisted even after 12 months of obturation, irrespective of the sealer and obturation technique used.

## 1 Introduction

The successful outcome of endodontic therapy relies heavily on the effective removal of bacteria during treatment, as well as the prevention of bacterial recolonization within the root canal (Siqueira Jr and Rôças 2008). However, despite meticulous chemo‐mechanical preparation (CMP), achieving complete eradication of microorganisms remains nearly impossible (Peters et al. 2001; Siqueira Jr and Rôças 2008; Kapralos et al. 2018). The root canal microbiome following CMP remains notably diverse, encompassing members from the phyla Firmicutes, Bacteroidetes, Actinobacteria, Proteobacteria, Fusobacteria, Spirochaetes, Synergistetes, and Saccharibacteria (Nardello et al. 2022). However, certain taxa demonstrate greater resilience to endodontic disinfection procedures, particularly those within the phylum Firmicutes, which includes streptococci and enterococci (Nardello et al. 2022).

Recognizing that the complete eradication of microorganisms during CMP is unrealistic, it is crucial that the obturation phase creates a durable seal that encloses any remaining microorganisms within the root canal system (Siqueira Jr and Rôças 2008). Due to the limited sealing capability of gutta‐percha, the incorporation of endodontic sealers is vital to effectively fill the irregularities in the root canal system and the dentinal tubules, thus trapping residual bacteria between the root cementum and the sealer (Arias‐Moliz et al. 2015; Kapralos et al. 2018).

Resin‐based sealers, such as AH Plus (Dentsply/De Trey GmbH, Konstanz, Germany), marketed as AH Plus Jet, are widely accepted by clinicians and supported by substantial evidence regarding their quality (Silva Almeida et al. 2017). In parallel, bioceramic sealers have gained increasing interest in the field of endodontics. These sealers are primarily composed of calcium silicates, which are known for their favourable physicochemical and biological properties. BioRoot RCS (Septodont, Saint‐Maur‐des‐Fossés, France) is noted for its high adhesion (Melo et al. 2023), effective penetration into dentinal tubules (Viapiana et al. 2016), and excellent biocompatibility (Camps et al. 2015; Siboni et al. 2017; Seo et al. 2019). Additionally, BioRoot RCS can induce hard tissue deposition (Prüllage et al. 2016; Siboni et al. 2017; Al‐Sabawi and Al‐Jubori 2023) and exhibits antimicrobial properties (Arias‐Moliz and Camilleri 2016; Alsubait et al. 2019).

With the ongoing development and enhancement of endodontic sealing materials, there has been a persistent effort to explore new protocols for applying substances and techniques aimed at improving treatment outcomes. Research has indicated that ultrasonic activation of sealers can expedite the setting reaction and enhance their mechanical properties (Silva et al. 2022; Jordani et al. 2024; Pauletto et al. 2024; Kim et al. 2024). Additionally, it can help reduce the presence of air bubbles within the material (Kleverlaan et al. 2004) and facilitate deeper penetration of sealers into dentinal tubules (Guimarães et al. 2014; Carneiro et al. 2023). However, there is a limited number of studies assessing the impact of ultrasonic activation on the antimicrobial efficacy of endodontic sealers (Alcalde et al. 2017; Viana et al. 2025).

Building on this background, the present ex vivo study aimed to evaluate the survival of bacteria organized in a dual‐species biofilm ( *Enterococcus faecalis* and *Streptococcus oralis* ) at both 30 days and 12 months following obturation with AH Plus Jet and BioRoot RCS sealers. To our knowledge, this is the first study in the literature to undertake such an evaluation. Additionally, the study sought to assess the impact of the antimicrobial activity of endodontic sealers, with and without ultrasonic activation, on bacterial viability in obturated canals. The null hypothesis posits that no microbial growth will be observed at either the 30‐day or 12‐month marks post‐filling, and that no differences will arise based on the type of filling cement used or the application of ultrasonic activation.

## 2 Materials and Methods

The manuscript of this laboratory study was prepared in accordance with the Preferred Reporting Items for Laboratory studies in Endodontology (PRILE) 2021 guidelines (Nagendrababu et al. 2021) (Figure 1).

**FIGURE 1:**
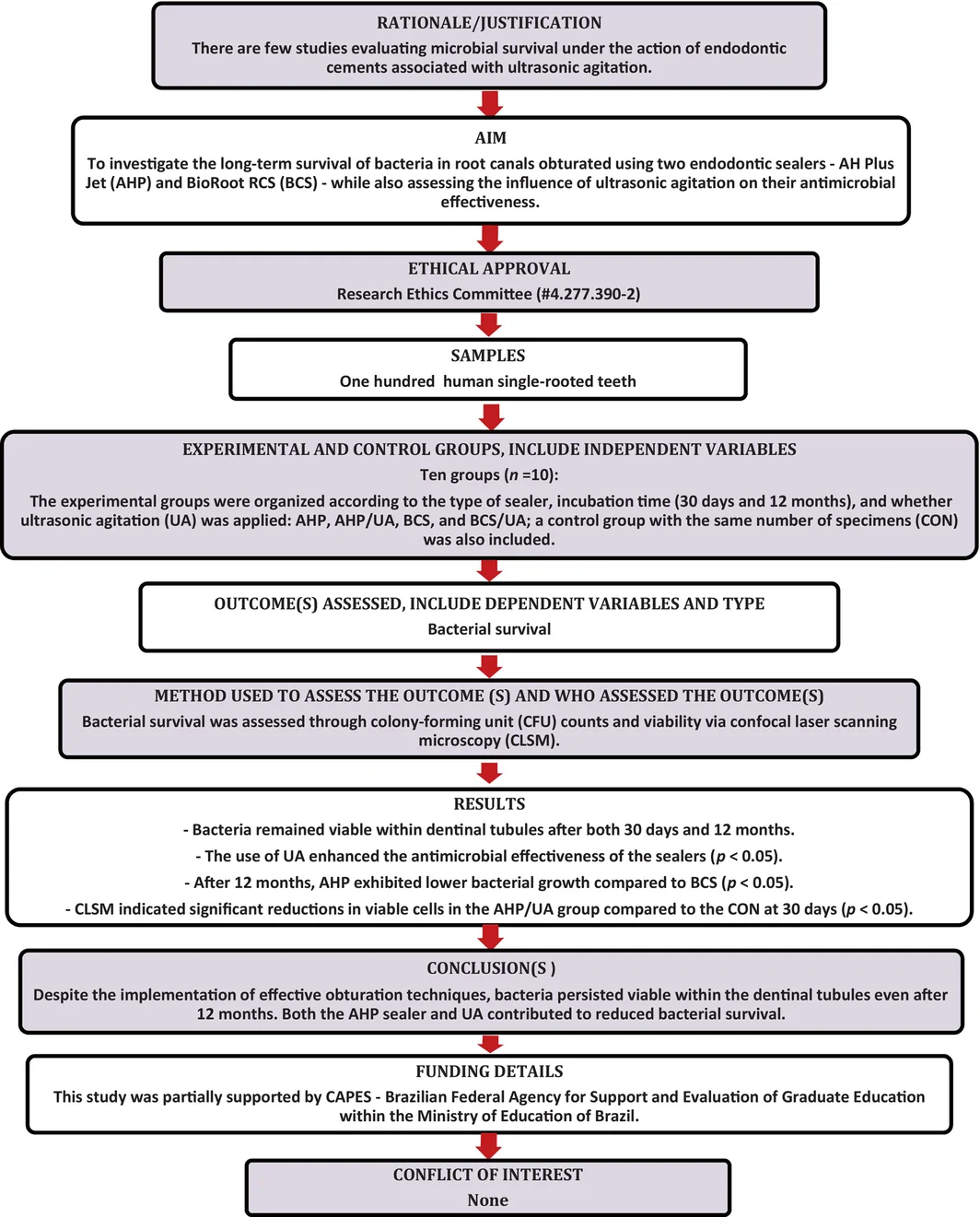
PRILE flowchart.

### 2.1 Microorganisms

Gram‐positive bacterial strains of *Enterococcus faecalis* (ATCC 29212) and *Streptococcus oralis* (PB 182) were used. Bacterial stocks were preserved in Brain Heart Infusion (BHI; Kasvi, São José dos Pinhais, PR, Brazil) medium containing 20% glycerol and stored at −80°C. For strain reactivation, 200 μL of bacterial extract were inoculated into tubes containing 9 mL of BHI broth and 1 mL of 10% glucose, in duplicate, and incubated at 37°C for 18 h. Culture purity was verified by Gram staining, and the inoculum was standardized to an optical density of 0.5 (620 nm), corresponding to a turbidity equivalent to 1–2 × 10^8^ CFU/mL.

### 2.2 Bacterial Survival Analysis and Sample Size Calculation

As this analysis required the use of extracted human teeth, the study protocol was submitted to and approved by the local Research Ethics Committee (#4.277.390‐2). Sample size calculation was performed using G*Power for Mac version 3.1 (Heinrich Heine University, Düsseldorf, Germany). Based on the results reported by Ioannidis et al. (2019), including mean values and standard deviations, the Wilcoxon–Mann–Whitney test was selected with an effect size of 0.75, alpha error probability of 0.1, and statistical power of 80% at a significance level of 5%, resulting in a minimum sample size of 10 specimens per group.

After ethics approval (Protocol #4.277.390), a total of 100 human single‐rooted teeth with straight roots, of patients between 18 and 45 years old, extracted for therapeutic reasons unrelated to this study, were selected. Preoperative periapical radiographs were taken in mesiodistal and buccolingual projections to select teeth with similar root canal mesiodistal and buccolingual diameter ratios. Teeth presenting root fractures, multiple canals, calcifications, oval‐shaped canals, curvatures, incomplete root development, resorptions, or previous endodontic treatment were excluded and replaced.

### 2.3 Specimen Preparation

Teeth were cleaned and stored in 0.1% thymol. Following the protocol described by Ioannidis et al. (2019), crowns were removed at the cemento‐enamel junction, standardizing root length at 13 mm up to the apical foramen. Initially, a size 15 K‐file (Dentsply/Maillefer, Ballaigues, Switzerland) was introduced into the root canal until its tip extended 1.0 mm beyond the apical foramen and became visible. The file was then retracted 2.0 mm to establish the working length (WL). The canal was flooded with 2.0 mL of 2.5% sodium hypochlorite (Biodinâmica Química e Farmacêutica, Ibiporã, PR, Brazil) and explored with a K‐file adjusted to the WL. All irrigation procedures were performed using a 10 mL disposable syringe and a 0.55 × 20 mm hypodermic needle (Becton Dickinson, Franklin Lakes, NJ, United States). Cervical preparation was performed with a CP Drill (Helse Ultrasonics, São Paulo, SP, Brazil), and canals were instrumented with Reciproc R25 instruments (size 25, 0.08 taper) (VDW GmbH, Munich, Germany) driven by a VDW Silver motor (VDW GmbH) up to the WL. The canals were irrigated with 2 mL of 2.5% sodium hypochlorite after each sequence of three pecking motions, with the irrigating solution delivered over a 30‐s period. Final irrigation consisted of 17% EDTA (Biodinâmica Química e Farmacêutica) for 3 min, followed by 5 mL of saline solution (Farmax, Jovelino Rabelo, MG, Brazil).

The root surfaces, including the apical foramen, were coated with two layers of red nail varnish to simulate clinical conditions and prevent the leakage of solutions, including irrigating solutions and culture media. The specimens were placed in Eppendorf tubes containing 1 mL of BHI broth; the tubes were sterilized in an autoclave at 121°C, 1 ATM, for 15 min. Samples were then incubated at 37°C for 24 h to confirm the absence of bacterial growth.

### 2.4 In Vitro Contamination of Specimens

Microbiological procedures and root manipulation were performed in a laminar flow cabinet. After overnight growth of both bacterial species ( *E. faecalis* and *S. oralis* ), culture purity was confirmed, and the bacterial suspension was adjusted to a McFarland standard of 0.5. Tubes containing roots immersed in sterile BHI were sonicated for 15 min to facilitate culture medium penetration into dentinal tubules. Subsequently, the BHI in each microtube was removed, and 100 μL of *E. faecalis* , 100 μL of *S. oralis* , 700 μL of sterile BHI broth, and 100 μL of sucrose were added. The eppendorf tubes containing the submerged roots were incubated at 37°C in 5% CO_2_ for 21 days to allow biofilm formation. Every 48 h, the BHI medium in each sample was replaced without adding new microorganisms.

### 2.5 Microbiological Sampling of the Specimens

After 21 days of incubation, the contaminated specimens were removed from the microtubes and placed on sterile filter paper in a laminar flow chamber. Before each sampling procedure, the canals were gently filed using sterile size 25 Hedstroem files (Dentsply/Maillefer), with three filing strokes performed over 30 s, followed by the insertion of three sterile size 25, 0.02 taper absorbent paper points (Dentsply/Maillefer), which remained inside the canal for 1 min. Each paper point was individually deposited in Eppendorf tubes containing 1 mL of sterile saline solution; this sample was designated as S1.

Following the initial sampling (S1), all specimens were reinstrumented. The canals were flooded with 10 mL of 2.5% sodium hypochlorite for 2 min and then prepared using Reciproc R40 instruments (size 40, 0.06 taper), which were introduced up to the working length (WL). During instrumentation, 2 mL of irrigating solution was delivered over 30 s after every three pecking motions. Upon completion of preparation, the canals were irrigated with 2 mL of 5% sodium thiosulfate (Formular, Sobral, CE, Brazil) for 1 min to neutralize the sodium hypochlorite. Subsequently, microbiological sampling of the canals was carried out with sterile size 40, 0.02 taper absorbent paper points as previously described; these samples were designated as S2.

After the post‐instrumentation sampling (S2), the canals were filled with 0.5 mL of 17% EDTA for 3 min, followed by irrigation with 5 mL of sterile saline solution for 1 min, and dried with sterile size 40, 0.02 taper absorbent paper points. The teeth were numbered and randomly allocated (based on bacterial count) into 10 groups (*n* = 10) using online software (http://www.openepi.com/menu/openepimenu.htm). The endodontic sealers AH Plus Jet and BioRoot RCS, whose compositions are listed in Table 1, were used.

**TABLE 1:** Sealers composition.

	AH Plus Jet (Dentsply/De Trey GmbH)	BioRoot RCS (Septodont)
Paste A/Powder	Bisphenol‐A epoxy resin	Tricalcium silicate
	Bisphenol‐F epoxy resin	Povidone
	Calcium tungstate	Zirconium oxide
	Zirconium oxide	
	Silica	
	Iron oxide pigments	
Paste B/Liquid	Dibenzyl diamine	Calcium chloride
	Aminoadamantane	Polycarboxylate
	Tricyclodecane diamine	Water
	Calcium tungstate	
	Zirconium oxide	
	Silica	
	Silicone oil	

The experimental groups were organized according to the type of sealer, incubation time (30 days and 12 months), and whether ultrasonic agitation (UA) was applied: AHP, AHP/UA, BCS, and BCS/UA; a control group with the same number of specimens (CON) was also included. Sealers were prepared according to the manufacturer's instructions and delivered into the root canals using a size 40 lentulo spiral (Dentsply/De Trey GmbH, Konstanz, Germany). In groups receiving UA, immediately after sealer placement, two 20‐s cycles of agitation in different directions (mesiodistal and buccolingual) (Carneiro et al. 2023) were performed with a smooth conical tip (E1/Irrisonic; Helse Ultrasonics, Santa Rosa do Viterbo, SP, Brazil) coupled to a piezoelectric ultrasonic device (Ultrawave Piezo Ultrasonic Scaler, Ultradent Products Inc., South Jordan, UT, USA) set to power level 3 (approximately 30%). Subsequently, standardized gutta‐percha cones from the Reciproc system (size 40, 0.06 taper) were inserted into the canals to WL. Coronal excess was removed with a heated instrument (2–3 mm below the cementoenamel junction), followed by vertical condensation with Schilder pluggers. The coronal seal was performed with light‐cured composite resin, following a sequence of 37% phosphoric acid etching for 15 s, application of Single Bond adhesive (3 M Dental Products), and placement of approximately 2 mm of resin (Filtek Z350; 3 M Dental Products).

In the control group, canals were filled solely with gutta‐percha cones, without endodontic sealer, and restored with composite resin. After obturation, all specimens were placed in vials containing 1.0 mL of sterile saline solution and incubated at 37°C and 5% CO_2_ for 30 days and 12 months. Before incubation, the external root surfaces and coronal access cavities were disinfected using a 2.5% sodium hypochlorite solution. This was followed by neutralization with a 5% sodium thiosulfate solution and rinsing with sterile saline. Each specimen was individually contained in a sealed vial, which was further secured with laboratory sealing film to maintain the integrity of the incubation environment and minimize the risk of external contamination during the experimental period. Throughout the 30‐day and 12‐month incubation durations, the specimens were periodically monitored for any signs of contamination by assessing medium turbidity, precipitate formation, and colour changes. Any specimen exhibiting these indicators was excluded from the study.

After the incubation period, canal filling materials were removed using a size 4 Gates‐Glidden drill in the coronal third and Reciproc R50 instruments (size 50, 0.05 taper) (VDW GmbH, Munich, Germany) in the middle and apical thirds, under constant irrigation with 5 mL of sterile distilled water. Digital periapical radiographs were used to confirm the removal of the obturation material. Once gutta‐percha was removed, a final irrigation was performed with 5 mL of the same solution. Microbiological sampling was then carried out as previously described; these samples were designated as S3.

After the S3 sampling, the roots were immersed in an Eppendorf tube containing 1 mL of sterile BHI broth, which served as a substrate for the survival of potential viable bacteria. The specimens were again incubated at 37°C and 5% CO_2_. After 7 days, a final microbiological sampling (S4) was performed to evaluate whether any bacteria remaining after treatment were able to survive and retain the ability to proliferate after removal of the filling material.

### 2.6 Dilution and Plating of Samples

Immediately after each collection, the Eppendorf tubes containing the used absorbent paper points were agitated for 30 s. A 10‐fold serial dilution was performed in saline solution, and 10 μL aliquots of each dilution were plated on BHI agar and incubated for 48 h at 37°C in 5% CO_2_ for total bacterial count, and on M‐*Enterococcus* agar to allow growth exclusively of *E. faecalis* colonies. Colony‐forming units (CFU) were counted after 48 h of incubation at 37°C in 5% CO_2_. The *S. oralis* bacterial count was calculated by subtracting the bacterial quantity on M‐*Enterococcus* agar from the total bacterial count. Bacterial colonies were counted manually by visual inspection of the agar plates under appropriate illumination by a trained evaluator blinded to the experimental groups. Considering the differences in bacterial load among the sampling periods, colony counting was performed using the dilution that provided countable numbers of colonies for each sample.

### 2.7 Cell Viability Analysis Using Confocal Laser Scanning Microscopy (CLSM)

After the final microbiological collection (S4), the contaminated specimens were sectioned longitudinally in the buccopalatal direction using a diamond disc in a precision cutting machine under refrigeration, following the protocol described by Alcalde et al. (2017). After sectioning, the samples were immersed in 17% EDTA for 5 min and rinsed with sterile saline solution. Specimens were stained for 10 min with 30 μL of the LIVE/DEAD BacLight Bacterial Viability Kit (Invitrogen Molecular Probes, Eugene, OR, USA) at room temperature. This viability kit contains two nucleic acid‐binding dyes: SYTO 9, which labels all bacteria in a population regardless of membrane integrity, and propidium iodide (PI), which penetrates cells with compromised or damaged membranes. When both dyes are present in cells with damaged membranes, SYTO 9 fluorescence is reduced due to displacement by PI staining. Bacteria with intact cell membranes fluoresce green, while those with damaged membranes fluoresce red.

After staining, the specimens were immersed in saline solution to remove excess dye, mounted on glass slides with immersion oil, and examined under CLSM (Leica TCS SPE; Leica Microsystems GmbH, Mannheim, Baden‐Württemberg, Germany) at 40× magnification. Images were acquired at 1 μm intervals with a resolution of 1024 × 1024 pixels. For each specimen, four superficial images (close to the root canal surface) and four deep images were obtained using Leica Application Suite‐Advanced Fluorescence software (LAS AF, Leica Microsystems GmbH). CLSM analysis was performed qualitatively to verify the presence of live (green fluorescence) and dead (red fluorescence) bacteria within the dentinal tubules. All image analyses were performed by a trained operator blinded to the experimental groups.

### 2.8 Statistical Analysis

Data were tabulated and subjected to the Shapiro–Wilk test to assess normality. Given the nonparametric distribution, Kruskal–Wallis analysis of variance and Dunn's multiple comparison test were used for intergroup comparisons under two factors (cements and ultrasonic agitation). The Mann–Whitney test was used for intragroup comparisons. Further statistical analysis was performed, also isolating the variables. Statistical significance was set at 5.0%.

## 3 Results

All specimens completed the experimental period and were included in the final analysis. Throughout the 30‐day and 12‐month incubation periods, there was no evidence of external contamination detected in any specimen, as indicated by the absence of medium turbidity, precipitate formation, or colour changes. The S1 samples (prior to preparation) demonstrated an average growth of 10^7^ cells, reflecting the reliability of the contamination protocol utilized. Additionally, the chemo‐mechanical preparation (S2) resulted in a significant reduction of bacterial counts in the specimens to 10^2^ cells (*p* < 0.05). The bacterial levels observed immediately after the removal of root filling (S3) remained comparable to those in the S2 samples. However, a notable increase in bacterial counts was recorded after 7 days of exposure to nutrition (S4), in comparison to S3 samples (*p* < 0.05) across all groups at both incubation periods, ultimately reaching contamination levels similar to those observed in S1 (Figure 2).

**FIGURE 2:**
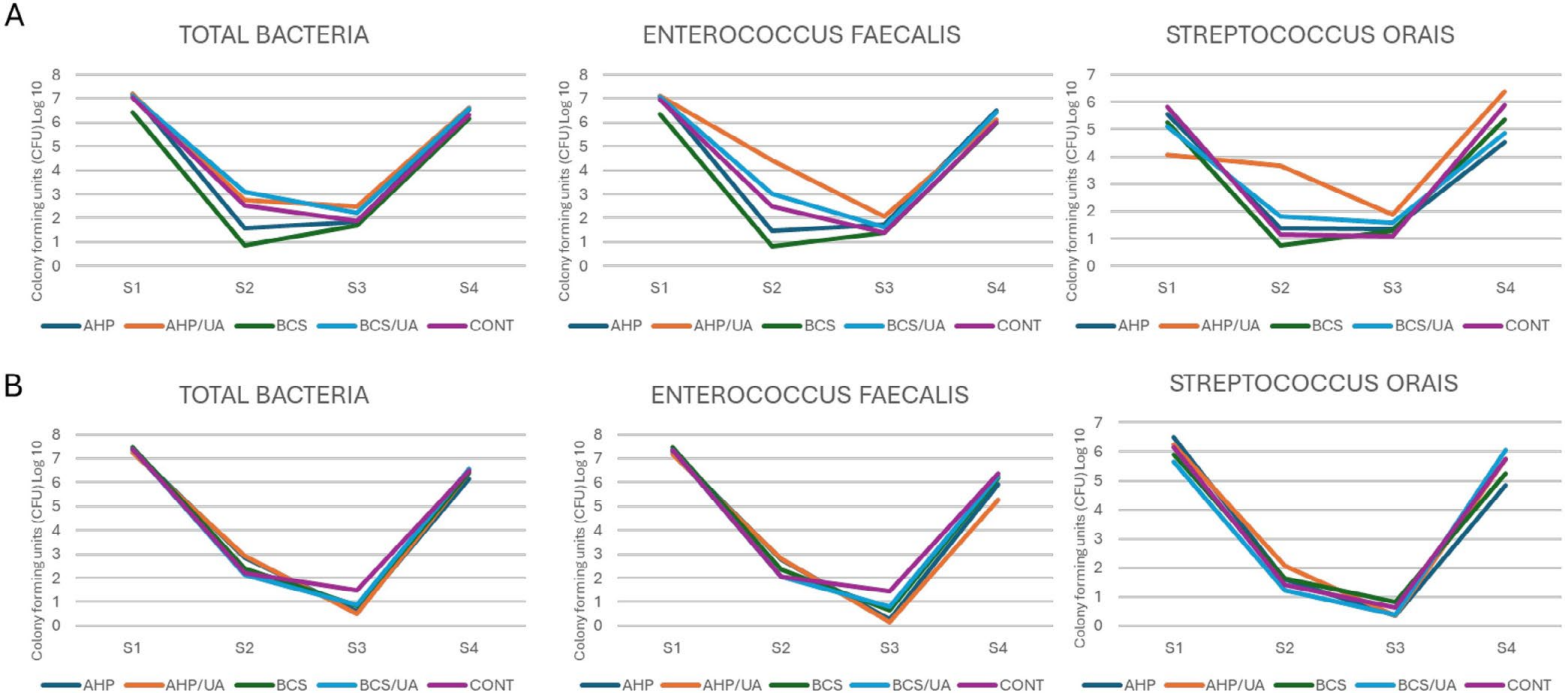
Graphs representing the count of CFU (log 10) from analyses performed over 30 days (A) and 12 months (B). Initial count (S1), post‐instrumentation (S2), after filling removal (S3), with additional samples after 7 days (S4).

Tables 2 and 3 present the log10‐transformed CFU counts in S3 and S4 samples for the 30‐day and 1‐year old obturations, respectively. Statistical analysis revealed no significant differences in total bacterial counts among the groups (*p* > 0.05) across all samples. However, when *E. faecalis* growth between S3 and S4 was compared, the AHP group demonstrated significantly lower bacterial growth than the BCS group during the 12‐month incubation period (*p* < 0.05) (Table 4). In terms of ultrasonic agitation (UA) use, UA was found to effectively reduce bacterial growth after 30 days of incubation (S4/S3) when compared to the control group (*p* < 0.05) (Table 4).

**TABLE 2:** Medians (min–max) of bacterial counts (log_10_) with a 30‐day incubation period immediately after root filling removal (S3) and after 7 days of nutritional exposure (S4).

Samples	AHP	AHP/UA	BCS	BCS/UA	CON
*S3*					
Total bacteria	2.09^a,A^	2.67^a,A^	1.84^a,A^	2.79^a,A^	1.87^a,A^
	(0.00–4.56)	(1.69–3.30)	(0.00–3.27)	(0.00–3.41)	(1.69–2.30)
*E. faecalis*	2.00^a,A^	2.17^a,A^	1.54^a,A^	2.19^a,A^	1.69^a,A^
	(0.00–4.47)	(0.00–3.00)	(0.00–2.82)	(0.00–3.46)	(0.00–1.87)
*S. oralis*	1.47^a,A^	1.87^a,A^	1.49^a,A^	1.90^a,A^	1.69^a,A^
	(0.00–3.81)	(0.00–3.00)	(0.00–3.26)	(0.00–3.14)	(0.00–2.07)
*S4*					
Total bacteria	6.55^a,B^	6.63^a,B^	6.43^a,B^	6.69^a,B^	6.22^a,B^
	(5.98–6.93)	(6.07–7.16)	(5.51–6.68)	(5.91–6.85)	(5.54–6.82)
*E. faecalis*	6.46^a,A^	6.27^a,A^	6.08^a,A^	6.61^a,A^	5.87^a,A^
	(5.98–6.91)	(5.43–6.64)	(5.86–6.71)	(5.86–6.71)	(5.00–6.66)
*S. oralis*	5.45^a,A^	6.36^a,A^	5.54^a,A^	5.60^a,A^	5.95^a,A^
	(0.00–5.96)	(5.96–7.09)	(4.47–6.18)	(0.00–6.60)	(5.39–6.46)

*Note:* Lowercase superscript letters (a,b) indicate significant differences between groups within each incubation period. According to Kruskal–Wallis and Dunn's multiple comparison tests (*p* < 0.05). Uppercase superscript letters (A,B) indicate significant differences between collections within each group and incubation period. According to the Mann–Whitney test (*p* < 0.05).

Abbreviations: AHP, AH Plus Jet; AHP/UA, AH Plus Jet/Ultrasonic agitation; BCS, BioRoot RCS; BCS/UA, BioRoot RCS/Ultrasonic agitation; CON, Control group.

**TABLE 3:** Medians (min–max) of bacterial counts (log_10_) with a 1‐year incubation period immediately after the removal of root filling (S3) and after 7 days of exposure to nutrition (S4).

Samples	AHP	AHP/UA	BCS	BCS/UA	CON
*S3*					
Total bacteria	0.00^a,A^	0.00^a,A^	0.00^a,A^	0.69^a,A^	1.70^a,A^
	(0.00–2.00)	(0.00–1.39)	(0.00–2.82)	(0.00–2.24)	(0.00–2.60)
*E. faecalis*	0.00^a,A^	0.00^a,A^	0.00^a,A^	0.69^a,A^	1.70^a,A^
	(0.00–2.09)	(0.00–1.39)	(0.00–2.43)	(0.00–2.39)	(0.00–2.43)
*S. oralis*	0.00^a,A^	0.00^a,A^	0.00^a,A^	0.00^a,A^	0.00^a,A^
	(0.00–1.39)	(0.00–1.39)	(0.00–2.60)	(0.00–1.70)	(0.00–2.24)
*S4*					
Total bacteria	6.19^a,B^	6.35^a,B^	6.57^a,B^	6.57^a,B^	6.49^a,B^
	(0.00–6.55)	(6.20–6.68)	(5.75–6.71)	(6.30–6.76)	(6.24–6.62)
*E. faecalis*	5.86^a,B^	6.25^a,B^	6.32^a,B^	6.43^a,B^	5.40^a,B^
	(0.00–6.51)	(0.00–6.63)	(5.12–6.79)	(5.69–6.55)	(6.11–6.46)
*S. oralis*	5.42^a,B^	5.73^a,B^	5.93^a,B^	6.27^a,B^	5.83^a,B^
	(0.00–6.39)	(5.51–6.23)	(0.00–6.50)	(5.00–6.34)	(5.23–6.11)

*Note:* Lowercase superscript letters (a,b) indicate significant differences between groups within each incubation period. According to Kruskal–Wallis and Dunn's multiple comparison tests (*p* < 0.05). Uppercase superscript letters (A,B) indicate significant differences between collections within each group and incubation period. According to the Mann–Whitney test (*p* < 0.05).

Abbreviations: AHP, AH Plus Jet; AHP/UA, AH Plus Jet/Ultrasonic agitation; BCS, BioRoot RCS; BCS/UA, BioRoot RCS/Ultrasonic agitation; CON, Control group.

**TABLE 4:** Two‐criteria analysis in relation to cement and ultrasonic agitation in bacterial reduction S4/S3.

	Factors	df	*p*	Post‐test
30 days				
Total bacteria	Interaction Sealer × UA		< 0.05	
	Sealer	2	> 0.05	
	UA	2	< 0.05	BCS > control
*E. faecalis*	Interaction Sealer × UA			
	Sealer	2	> 0.05	
	UA	2	> 0.05	
*S. oralis*	Interaction Sealer × UA			
	Sealer	2	> 0.05	
	UA	2	> 0.05	
12 months				
Total bacteria	Interaction Sealer × UA			
	Sealer	2	> 0.05	
	UA	2	> 0.05	
*E. faecalis*	Interaction Sealer × UA		< 0.05	
	Sealer	2	< 0.05	AHP>BCS
	UA	2	> 0.05	
*S. oralis*	Interaction Sealer × UA			
	Sealer	2	> 0.05	
	UA	2	> 0.05	

*Note:* According to Mann–Whitney test (*p* < 0.05).

Abbreviations: AHP, AH Plus Jet; AHP/UA, AH Plus Jet/Ultrasonic agitation; BCS, BioRoot RCS; BCS/UA, BioRoot RCS/Ultrasonic agitation; CON, Control group; df, degree of freedom.

Figures 3 and 4 present representative CLSM images of bacterial viability on superficial and deep dentine, captured 7 days after the removal of root filling (S4), for 30‐day and 1‐year‐old obturations, respectively. Overall, the CLSM findings corroborated the culture‐based results. In agreement with the CFU findings, CLSM images obtained after 30 days demonstrated reduced bacterial viability within the dentinal tubules following ultrasonic agitation of the sealers, particularly in the AHP/UA group (Figure 3B,G). In this group, a greater number of cells within the dentinal tubules were stained red, indicating the presence of dead bacteria, with this finding being more pronounced in the deep dentine (Figure 3G). Likewise, after 12 months, both the AHP (Figure 4A,F) and AHP/UA (Figure 4B,G) groups exhibited lower cell viability compared to the other groups.

**FIGURE 3:**
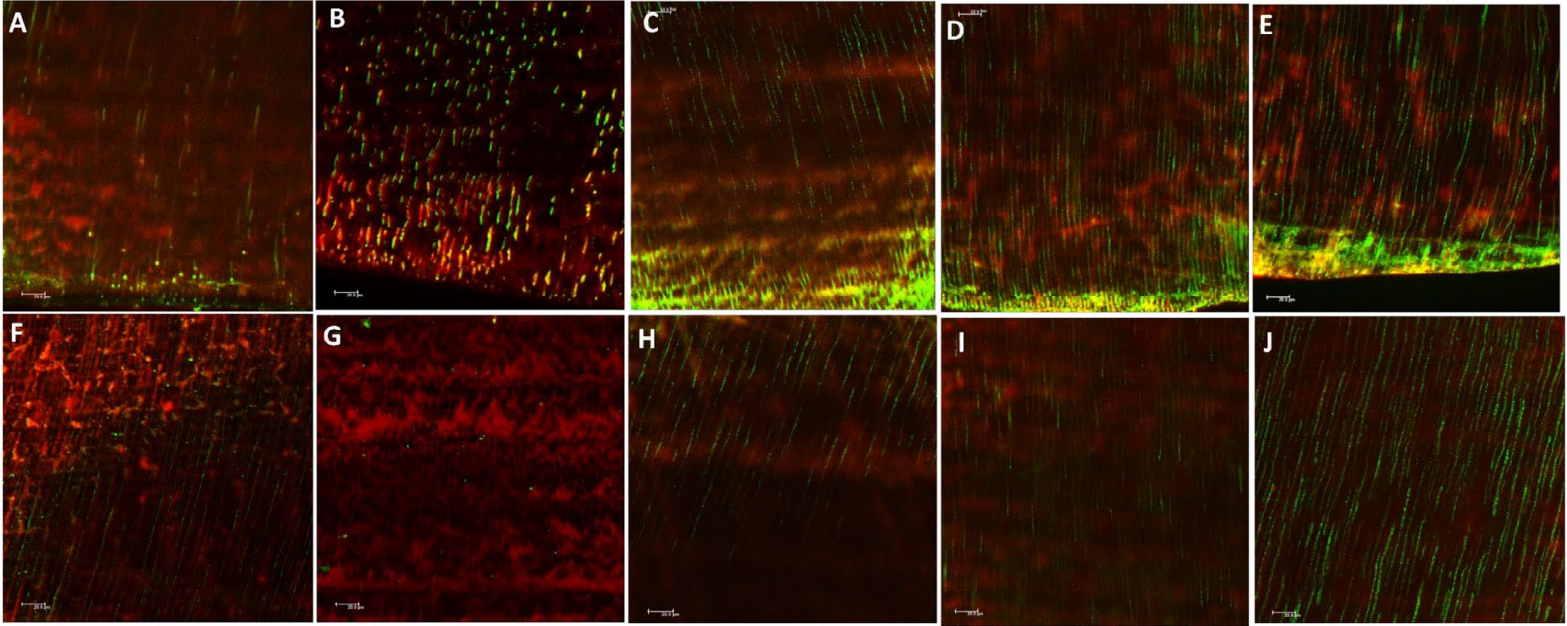
Representative images illustrating bacterial viability in dentine samples 7 days after the removal of root filling, in 30‐day‐old obturation, were obtained from the superficial (A, B, C, D, E) and deep (F, G, H, I, J) dentine using Confocal Laser Scanning Microscopy (CLSM). The samples were stained with the LIVE/DEAD BacLight Bacterial Viability Kit: A and F (AHP), B and G (AHP/AU), C and H (BCS), D and I (BCS/AU), and E and J (CON). In these images, red coloration signifies dead bacteria, while green indicates live bacteria. All images were captured at a magnification of 40×.

**FIGURE 4:**
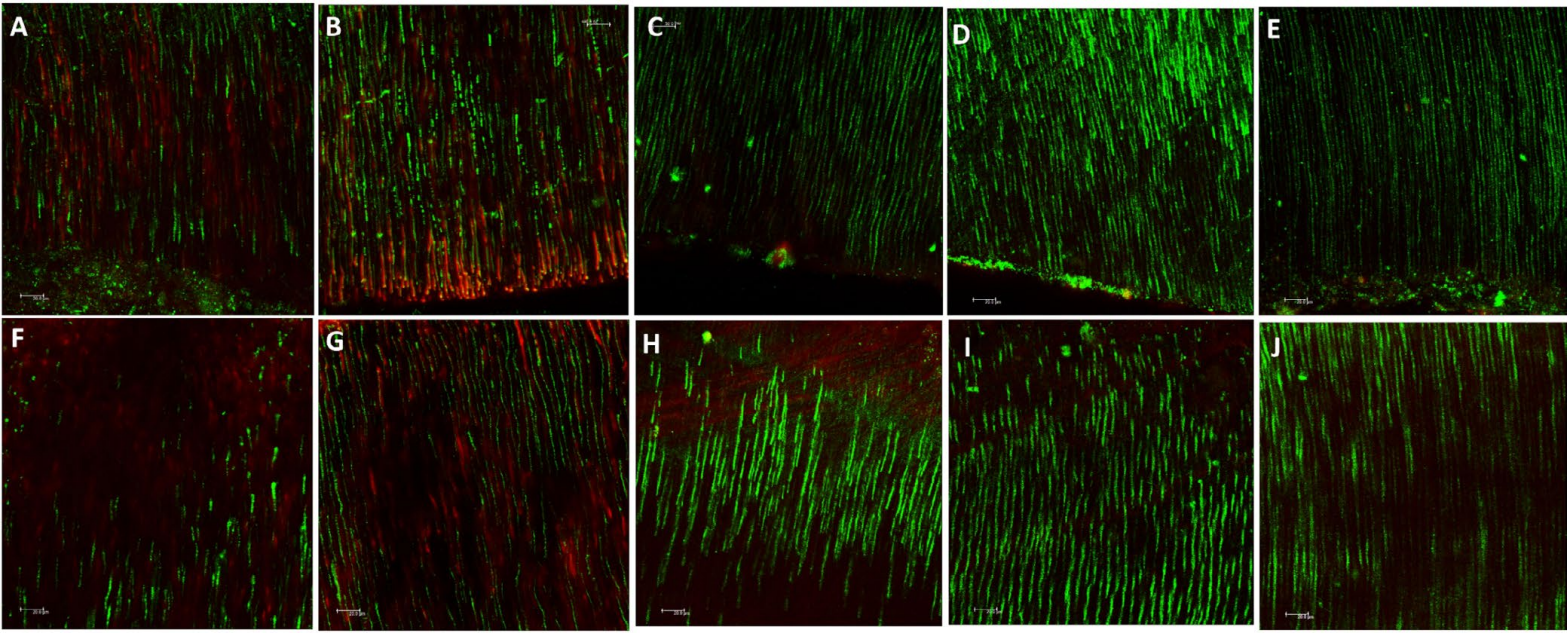
Representative images illustrating bacterial viability in dentine samples 7 days after the removal of root filling, in 1‐year‐old obturation, were obtained from the superficial (A, B, C, D, E) and deep (F, G, H, I, J) dentine using Confocal Laser Scanning Microscopy (CLSM). The samples were stained with the LIVE/DEAD BacLight Bacterial Viability Kit: A and F (AHP), B and G (AHP/AU), C and H (BCS), D and I (BCS/AU), and E and J (CON). In these images, red coloration signifies dead bacteria, while green indicates live bacteria. All images were captured at a magnification of 40×.

## 4 Discussion

The present study evaluated the long‐term survival of bacteria in root canals obturated with two distinct types of root canal sealers: the resin‐based sealer AH Plus Jet and the bioceramic sealer BioRoot RCS. The null hypothesis was rejected, as bacteria were found to persist within dentinal tubules both 30 days and 12 months after obturation, regardless of the sealer type used. These findings are consistent with previous research indicating that *E. faecalis* can remain viable in root canals for up to 12 months in the absence of nutrients (Sedgley 2007). The ability of enterococci to enter a viable but non‐cultivable state is considered a critical mechanism for their survival within the microenvironments of the root canal system (Sedgley et al. 2005; de Castro Kruly et al. 2022). These results highlight the importance of promptly performing a definitive coronal restoration post‐endodontic treatment, as well as the necessity of employing a proper obturation technique aimed at effectively sealing the root canal system.

In this context, the current study also aimed to investigate the impact of ultrasonic agitation on the antimicrobial efficacy of endodontic sealers. The second hypothesis was similarly rejected, as ultrasonic agitation significantly enhanced the antibacterial activity of the endodontic sealers during the 30‐day period. This improvement may be associated with improved adaptation and deeper intratubular penetration of the sealers (Alcalde et al. 2017). Such penetration may occlude the entrances of the dentinal tubules, thereby reducing the likelihood of bacterial re‐entry into the main canal. In addition to facilitating sealer penetration, ultrasonic agitation may also influence the pH of the materials, potentially enhancing their initial antimicrobial properties (Viana et al. 2025). The mechanisms through which ultrasonic activation affects the pH of endodontic cements remain not fully understood. One explanation may be the improved mixing of the materials, which could enhance interactions among their constituents and promote greater release of active components (Viapiana et al. 2014). However, the antimicrobial effect was not maintained after 12 months, when similar bacterial levels were observed in the S3 and S4 cultures among all experimental groups, irrespective of the sealer used or the application of ultrasonic agitation. These findings suggest that the antimicrobial benefits associated with ultrasonic activation may be limited to the early period after obturation.

Nevertheless, the findings of the present study should be interpreted with caution, since one of its limitations was the use of a dual‐species biofilm model, which does not fully reflect the polymicrobial complexity of endodontic infections. *Enterococcus faecalis* and *Streptococcus oralis* were selected as representative members of the phylum Firmicutes, which are frequently detected in the root canal microbiome both before and after chemo‐mechanical preparation (Nardello et al. 2022). Additionally, research indicates that these species can infiltrate dentinal tubules and remain viable for extended periods (AlShwaimi et al. 2016; Love et al. 1997; Stauffacher et al. 2017; Saleh et al. 2004; Hahn and Hanford 2021). Despite the limited diversity of species, this study effectively monitored the growth of these bacteria at various stages of treatment: the initial sample prior to treatment (S1), the post‐instrumentation sample (S2), and postoperative samples taken immediately after root‐filling removal (S3), and 7 days later (S4). Notably, the S4 samples indicated that bacteria were able to proliferate in the nutrient‐rich environment following the removal of the filling material. The identification of these species both at the beginning of treatment and after root filling removal supports the model of contamination in dentinal tubules and the persistence of these species after obturation.

In this study, we observed significant growth of both bacterial strains in the S1 samples prior to treatment. The chemo‐mechanical preparation resulted in a considerable reduction in the number of bacterial cells in the S2 samples, whereas no significant differences were noted when comparing S2 to S3 samples. In contrast, a notable increase in bacterial counts was recorded after 7 days (S4), showing that persisting bacteria were able to proliferate once nutrient conditions were reestablished. Clinically, these results underscore the importance of obturation procedures, as they have proven effective—regardless of the material used—in containing contamination within the dentinal tubules, with canal lumen contamination levels comparable to or lower than those achieved through chemo‐mechanical preparation. This aspect has been extensively discussed and recognized as a crucial factor influencing the success of endodontic treatment (Siqueira Jr and Rôças 2008).

Bacterial survival was analysed by comparing bacterial growth between S3 and S4 samples. In addition to employing culture‐based methods, Confocal Laser Scanning Microscopy (CLSM) was utilized to confirm the presence and viability of bacteria within the dentinal tubules seven days after the removal of fillings under nutrient conditions. CLSM images revealed a significant number of viable bacteria after both 30 days and 12 months following obturation. These CLSM findings corroborate the results from CFU counts, demonstrating substantial bacterial growth after nutrient exposure. The culture‐based results showed that, after 30 days, UA effectively reduced bacterial growth compared to the control group. Consistent with the CFU findings, CLSM images obtained after 30 days also demonstrated that ultrasonic agitation of the sealers reduced bacterial viability within the dentinal tubules compared to the control group, particularly in the AH Plus/UA group, where a marked predominance of red‐stained cells was observed. These findings are in line with the findings of Alcalde et al. (2017), who noted a beneficial effect of ultrasonic agitation on the quality of root canal fillings, improved intratubular penetration, and increased antimicrobial activity of AH Plus.

After 12 months, the culture‐based analysis demonstrated that AH Plus Jet exhibited superior antimicrobial activity against *E. faecalis* compared to BioRoot RCS. Supporting the CFU findings, CLSM images also revealed that cell viability was lower in both the AHP and the AHP/UA groups compared to the BCS and control groups. These findings may be attributed to AH Plus's ability to penetrate more deeply into the dentinal tubules than bioceramic cements, such as BioRoot RCS (Arikatla et al. 2018; Kim et al. 2019), thereby diminishing bacterial survival. AH Plus demonstrates low solubility after setting, which results from amine groups in its components reacting with epoxy groups to create a strong covalent bond that effectively seals the dentinal tubules (Borges et al. 2012). Furthermore, the antimicrobial activity of AH Plus has been attributed to the toxicity of certain unpolymerized components, including unreacted epoxy resin and amines (Kayaoglu et al. 2005; Wainstein et al. 2016), which maintain a residual antimicrobial effect within the dentinal tubules. In contrast, regarding the antimicrobial activity of BioRoot RCS, although several studies have highlighted the excellent antimicrobial properties of BioRoot RCS in both direct and indirect contact with biofilms (Long et al. 2020; Kapralos et al. 2021; Bose et al. 2020), this study revealed that its antimicrobial effect against *E. faecalis* was less pronounced than that of AH Plus Jet. Differences in biofilm composition (including species and maturation), variations in obturation techniques, and methodological factors related to observation time and approaches may explain the discrepancies observed across different studies.

The lack of significant differences in bacterial viability and microbial counts between canals obturated with sealer plus gutta‐percha and those obturated with gutta‐percha alone suggests that effective sealing of the root canal system may be more critical in limiting residual microbial growth than the intrinsic antimicrobial properties of the obturation materials. Once sealed, the intraradicular environment becomes nutritionally restricted, which may impair bacterial proliferation while allowing the persistence of viable microorganisms in a low‐metabolic state. Consequently, the similar microbial outcomes observed across the different obturation techniques may be attributed to nutrient deprivation and bacterial entombment, rather than variations in the antimicrobial properties of the materials employed (Sundqvist et al. 1998; Love 2001; Nair 2006). These findings reinforce the concept that nutrient restriction within the sealed canal system plays a pivotal role in controlling persistent intraradicular infection. However, these findings cannot be directly extrapolated to clinical conditions, since the present ex vivo model used a dual‐species biofilm under limited nutrient availability and in teeth with relatively simple root canal anatomy, whereas clinical endodontic infections involve more complex polymicrobial biofilms and intricate anatomical structures, including isthmuses, ramifications, and accessory canals, in which sealers play an essential role in preventing nutrient access and bacterial persistence.

Taken together, the present results indicate that, irrespective of the endodontic sealer used or the application of ultrasonic agitation, bacteria were able to remain viable within dentinal tubules for up to 12 months after treatment and obturation. Moreover, the ability of these microorganisms to proliferate once favourable nutritional conditions were reestablished reinforces the notion that microbial persistence may be primarily governed by environmental restriction. From a clinical perspective, these findings highlight the importance of immediate and effective coronal restoration following root canal treatment, as well as obturation techniques that maximize the long‐term sealing of the root canal system to entomb residual bacteria within the canal space. Finally, the persistence of viable microorganisms despite obturation emphasizes the need for further research into adjunctive strategies aimed at improving root canal disinfection. Future clinical studies should also investigate the critical bacterial load required to establish persistent or secondary infections after root canal treatment.

## 5 Conclusion

In light of the limitations of this study, it can be concluded that ultrasonic agitation reduced the ability of residual bacteria to recolonize the root canal space during the initial 30‐day period. However, viable bacteria persisted even after 12 months of obturation, irrespective of the sealer and obturation technique used, suggesting a limited long‐term antimicrobial effect of the sealers in contaminated root canals.

## Author Contributions

**F. L. P. Viana:** conceptualization, methodology, visualization, investigation, data curation, formal analysis, writing – original draft, writing – review and editing, project administration. **M. A. F. Damasceno:** methodology, investigation. **M. P. Alcalde**, **R. R. Vivan**, **M. A. H. Duarte:** methodology, visualization, investigation, data curation, writing – review. **I. G. de Moraes (in memoriam):** conceptualization. **E. T. Pinheiro**, **B. C. Vasconcelos:** conceptualization, methodology, visualization, investigation, data curation, formal analysis, writing – review and editing, supervision, project administration.

## Funding

This study was partially supported by CAPES—Brazilian Federal Agency for Support and Evaluation of Graduate Education within the Ministry of Education of Brazil.

## Disclosure

The authors have nothing to report.

## Conflicts of Interest

The authors declare no conflicts of interest.

## Data Availability

The data that support the findings of this study are available from the corresponding author upon reasonable request.
